# Supplementary material for: GoM DE: interpreting structure in sequence count data with differential expression analysis allowing for grades of membership
Source: Genome Biol. 2023 Oct 19;24:236. doi: 10.1186/s13059-023-03067-9 (PMC10588049; doi:10.1186/s13059-023-03067-9)
Supplement: Supplementary file 8 — Additional file 8. Review history. Document with the review history. [file 13059_2023_3067_MOESM8_ESM.docx]

**1^st^ round**

**Reviewer 1**

The authors proposed a new method to interpret the individual dimensions of a parts-based representation and illustrated the method in several single-cell RNA-seq and ATAC-seq data sets including simulated data. The tool is important for the community for the single cell data analysis. The manuscript is clearly written, and the authors provided source codes and data. I would recommend the publication if the following points are addressed.
1. With the growth of multi-omics and spatial transcriptomics, it is interesting to indicate whether it's possible, and if possible how well the method performs on datasets containing both ATAC and RNA-seq, or spatial transcriptomics datasets.
2. The authors made discussion on K selection. This is also one problem in clustering methods. As a recent paper could decide optimal cluster number by hierarchical clustering [eg. PMID: 35524494], is it possible to decide some criteria to automatically judge an optimal K.
3. As the batch effect is an important factor in single cell data, it will be illustrative to evaluate the performance on batched datasets in addition to single dataset.

**Reviewer 2**

The paper by Carbonetto et al introduces a new method to perform differential expression analysis in topic models. In particular, this approach accounts for the case where each cell can have fractional membership in each cluster. The novel contribution of the paper is an efficient MCMC-based computation of the least extreme LFC metric in the Poisson NMF model. While we believe that this will be a useful tool for the single-cell community, we make several suggestions that we believe will improve the quality of the manuscript. Overall, we believe that this paper has the potential to be of sufficient impact and scientific rigor for the journal, although we recommend further improvements as outlined below.


Major Comments:


Overall motivation


We believe that this is a potentially impactful contribution that would benefit from better motivation and context for the problem at hand. We have several unanswered questions after reading the paper/introduction, including: to what extent do other researchers compute differential expression across topics in single-cell data? If so, what are the primary methods/approaches that are used? If not, what are the main motivations / benefits that can be obtained by developing a method to compute differential expression across topics? Pinpointing these questions would help orient the reader and increase the impact of the paper.


Robustness to model assumptions


In the case where cells do actually belong to discrete cell types this method is expected to perform worse than the standard approach of clustering followed by DE analysis. Some simulations or real data should be included to demonstrate that the model can still perform well in this setting.


Authors should investigate the extent to which topics can learn noise and potentially create a simulation on single-topic data to measure the amount of noise for this approach.


Undetermined sign


We are concerned about the inclusion of the absolute value in the least extreme LFC metric (equation 6). This metric makes the most sense when the topic has the highest (or lowest) gene expression for a given gene. However, we are uncertain how to interpret this metric when a gene is of intermediate expression. For example, consider the case where there are three topics, A, B, and C of log-gene-expression 0, 2, and 4, respectively. According to this metric, the le-LFC of topic B would be 2, which could indicate a moderately strong differential expression. However, in reality, one would not consider this gene to be differentially expressed in topic B. In general, we would not consider a gene to be usefully differentially expressed within a topic if it's gene expression lies in the center of the gene expression distribution across topics.


There is also a related issue of how sign is determined for this metric. We don't believe that the method for determining sign was explained in this paper, and we recommend making this adjustment. However, the signed le-LFC metric appears to be used in the figures. We assume that the sign is related to the sign of the next closest topic. But we are not sure how the definition occurs in the Bayesian setting of multiple states. In any case, it seems hard to determine a sign in cases such as the example above for topic B.


Comparison to Frequentist method


While we believe DE across topics to be a worthwhile endeavor, we believe that the paper would be strengthened by better motivating the use of a Bayesian method for computing le-LFC. For example, one can consider simpler DE metrics computed directly based off the MLE or based off of MLE + fisher information matrix. It would be great if the authors could provide additional analyses that clearly demonstrate the necessity of the Bayesian approach.


Length of paper / number of datasets


Since this is primarily a computational methods paper, and Genome biology is a journal targeted at evaluating new methods, we wonder if the presentation of the biology of multiple datasets goes beyond what is necessary and could be reduced. Figures 6, 7, and 8 feel a little repetitive with some of the earlier figures, and we wonder if some can be moved to the supplement. In particular, the ATAC-seq data is probably the most interesting to focus on because it is application to a new type of data. We leave this as a suggestion for the discretion of the editor and the authors.


Minor Comments:

Appears to be no benchmark for runtime. For instance, how long did it take to obtain the results for Figure 1?

Page 14 line 45 add definition of highest posterior density (HPD) interval

For the analysis on simulated data (page 4 line 38) the number of cells is only 200, which is significantly smaller than typical single-cell RNA-seq data.

On Page 12, the section "Two group model" introduces new notation that could have been described using equation 9. Also, it appears that this model is never actually used in any other parts of the paper.

For figure 2, should include an equation (in methods or results) for the posterior z score.

Page 14 line 45 add definition of highest posterior density (HPD) interval

For the analysis on simulated data (page 4 line 38) the number of cells is only 200, which is significantly smaller than typical single-cell RNA-seq data.

Is this paper looking for DE across topics, or looking for changes in topic-level expression across populations? Should make this distinction more clearly at the start. I believe that it only does DE across topics.

We recommend citing the paper "Cell type-specific inference of differential expression in spatial transcriptomics" (Nature Methods, 2022) as a similar model in a different context.

Figure 1c: enrichment coefficients are less than 1. Are these in log space? If so, this could be more clearly clarified.

Typo: "genes upregulated in CD34+ in hematopoetic cells"

Figure 2: missing color scale?

Can the le-LFC metric also be applied to single cell RNA-seq data? Has it been applied before? If so, how well does it work?

The notation in the two groups model in the methods section does not match the earlier notation. Authors should stick to the l ('ell') variable or at least define the new variables in terms of the l variable. Also, the authors should state explicitly that they are considering discrete topics / non admixed for the two groups model because we found this to be confusing.

"Maximum a posteriori estimation": how are the gamma prior parameter values determined / motivated?

Some of the estimated DE values are extremely large (13-15). It would be good to present some plots based on the raw data that can convince the reader that these are real effects present in the data, rather than an artifact of the topic learning procedure.

**Authors’ response**

We thank the Editor and the reviewers for their feedback on our manuscript. We made several changes to the manuscript to address the reviewers’ comments, which we summarize here:

1. We added a paragraph to better motivate the MCMC algorithm (see “Quantifying uncer tainty and stabilizing LFC estimates” in Methods).

2. We added a table (Table 1) summarizing the running times for the GoM DE analyses for all 4 data sets.

3. We removed the “two-group model” from the paper since it was not essential and, as Reviewer 2 pointed out, might cause confusion.

4. We added a paragraph to the Discussion on the potential for applying topic modeling to “multi-omics” data.

5. We wrote an expanded discussion on dealing with batch effects.

6. Finally, we provided better theoretical justification for the use of the multinomial model for scATAC-seq data (see “Models for single-cell ATAC-seq data” in Methods). Although this was not requested by the reviewers, in revisiting the manuscript we believed that this was a methodological gap that needed to be addressed. In the new text, we explain that using the topic model for scATAC-seq data implicitly assumes *a multinomial model of the (binarized) accessibility data.* We show that this multinomial model is nearly equivalent to the binomial model that was previously suggested by Ashuach et al. (2022). In summary, we now provide a theoretical justification for the topic model that was missing from our earlier submission (and was missing from González-Blas et al. 2019).

In addition to these changes, we made other smaller changes in the manuscript to address the reviewers’ comments. All non-trivial changes have been highlighted in blue. For completeness, we include the remarks from the Editor in this letter.

Reviewer #1

The authors proposed a new method to interpret the in dividual dimensions of a parts-based representation and illustrated the method in several single-cell RNA-seq and ATAC-seq data sets including simulated data. The tool is important for the community for the single-cell data analysis. The manuscript is clearly written, and the authors provided source codes and data. I would recommend the publication if the following points are addressed.

Our response. Thank you for reviewing our manuscript and for the constructive feedback on our work. We have responded to the reviewer’s individual comments in the paragraphs below.

Comment 1. With the growth of multi-omics and spatial transcriptomics, it is interesting to indicate whether it’s possible, and if possible how well the method performs on datasets containing both ATAC and RNA-seq, or spatial transcriptomics datasets.

Our response. Following the reviewer’s suggestion, we have added a paragraph to the Discussion touching on the potential for applying topic modeling approaches to multimodal single-cell data such as sci-CAR (Cao et al., 2018). This is what we wrote:

Recently developed technologies profile both transcription and chromatin accessibility in single cells (Ma et al., 2020; Cao et al., 2018). For such data, one could fit two topic models, one to the RNA seq data and another to the ATAC-seq data. (With a careful initialization of the topic model fitting algorithm, the topics could be made more consistent across the two modalities.) But it would be preferable to analyze the multimodal data jointly for improved accuracy (Stuart and Satija, 2019; Shiga et al., 2021; Zhang et al., 2012; Yang and Michailidis, 2015; Jin, Zhang and Nie, 2020; Hao et al., 2023). Potentially, the strategy used in MOFA (Argelaguet et al., 2018, 2020) could be adapted for topic modeling—that is, the transcripts and accessibility profiles would share the same topic proportions, L, but each modality would have a different F. However, it remains to be seen how well this strategy works in practice.

For spatial transcriptomics data, we agree with the reviewer that there is the potential to apply topic modeling approaches to recover spatial (and other) structure from these data. However, we prefer not to discuss this in the manuscript since (a) there are already many papers on methods for spatial transcriptomics data; (b) there are subtleties in interpreting the results that may depend on the specifics of the technology. For example, for spatial transcrip tomic assays with lower resolution (e.g., 10 cells or so per location), topic proportions might be confounded with cell proportions. Given these points, we believe our comments would be too speculative.

Comment 2. The authors made discussion on *K* selection. This is also one problem in clus tering methods. As a recent paper could decide optimal cluster number by hierarchical clus tering (Zeng et al., 2022), is it possible to decide some criteria to automatically judge an optimal *K*.

Our response. There have been many papers written on selection of *K* in topic models and related models, including STRUCTURE, non-negative matrix factorization, and mixture models. Many of these papers have suggested different criteria for determining which *K* is “best”. Our view, which reiterates what we said previously in the Discussion, is that there is no single “optimal” *K*; a good choice of *K* will depend on the aims of the researcher, and in some cases it may be beneficial to show topic modeling results for several choices of *K*. For example, increasing the number of topics in the Mouse sci-ATAC-seq Atlas data reveals more structure within tissues; see https://tinyurl.com/2p99swdk.

The approach suggested by Zeng et al. (2022) is based on the idea that there is a “correct” number of underlying cell types. But the truth is almost always more complicated; consider that our notions of “cell type” do not fully account for underlying dynamic, spatial and other continuous processes shaping cells (Morris, 2019; Levsky and Singer, 2003).

Comment 3. As the batch effect is an important factor in single cell data, it will be illustrative to evaluate the performance on batched datasets in addition to single dataset.

Our response. The two scATAC-seq data sets illustrated the impact of batch effects on the topics; the Mouse Atlas data includes replicates for several tissues (Figure 7A), and the hematopoietic system data includes cells from multiple donors (Figure S15). We also had a paragraph in the Discussion about how to deal with batch effects in topic modeling analy ses of single-cell data. This being said, we agree with the reviewer that this is an important aspect of the analysis, so we have expanded on the discussion of batch effects in the revised manuscript.

Reviewer #2

We believe that this is a potentially impactful contribu tion that would benefit from better motivation and context for the problem at hand. We have several unanswered questions after reading the paper/introduction, including: to what extent do other researchers compute differential expression across topics in single-cell data? If so, what are the primary methods/approaches that are used? If not, what are the main motiva tions/benefits that can be obtained by developing a method to compute differential expression across topics? Pinpointing these questions would help orient the reader and increase the im pact of the paper.

Our response. Thank you very much for the detailed commentary on our work. We begin by answering your initial questions:

• *To what extent do other researchers compute differential expression across topics in single cell data? If so, what are the primary methods/approaches that are used?* To our knowl edge, this is has not been done previously because *no methods have been developed to do this.* In the Results (“Evaluation of DE analysis methods using simulated data“) we stated, “Since existing DE methods cannot handle the situation in which there are partial mem berships to groups, we mainly sought to verify that the method behaves as expected in the ideal setting when data sets are simulated from the topic model.” To our knowledge, the only method that we can compare to is the K-L divergence measure that was used in Dey, Hsiao and Stephens (2017) to identify “driving genes” for topics. However, this measure does not properly quantify uncertainty in expression differences, and therefore does not provide a measure of support for differential expression comparable to existing methods such as edgeR or DESeq2.

• *If not, what are the main motivations/benefits that can be obtained by developing a method to compute differential expression across topics?* We provided motivation in the abstract and introduction. In the introduction, we cited several papers that have use topic models (and related NMF methods) to analyze single-cell data. The focus of our paper is therefore the question of how to interpret the individual dimensions of a parts-based representation learned by fitting a topic model.

Comment 1: Robustness to model assumptions. In the case where cells do actually belong to discrete cell types this method is expected to perform worse than the standard approach of clustering followed by DE analysis. Some simulations or real data should be included to demonstrate that the model can still perform well in this setting.

Authors should investigate the extent to which topics can learn noise and potentially create a simulation on single-topic data to measure the amount of noise for this approach.

Our response. The topic model includes discrete clusters as a special case—topic *k* is a discrete cluster if the topic proportions *l_ik_* are all binary—so we wouldn’t necessarily expect performance to be worse in this setting. In any event, this is precisely the setting we consid ered in the first part of our simulations: “We began our evaluation by first considering the case of two groups in which there is no partial membership to these groups; that is, when the cells can be clearly separated into two cell types.” In these simulations, we compared the GoM DE analysis to two methods, DESeq2 and MAST, that assume discrete clusters. In particular, the DESeq2 and GoM DE methods, which are based on similar models, achieved very similar performance in this setting (Figures 4 and S7). Please see “Evaluation of DE analysis methods using simulated data” for more details.

Comment 2: Undetermined sign. We are concerned about the inclusion of the absolute value in the least extreme LFC metric (equation 6). This metric makes the most sense when the topic has the highest (or lowest) gene expression for a given gene. However, we are un certain how to interpret this metric when a gene is of intermediate expression. For example, consider the case where there are three topics, A, B, and C of log-expression 0, 2, and 4, re spectively. According to this metric, the l.e. LFC of topic B would be 2, which could indicate a moderately strong differential expression. However, in reality, one would not consider this gene to be differentially expressed in topic B. In general, we would not consider a gene to be usefully differentially expressed within a topic if it’s gene expression lies in the center of the gene expression distribution across topics.

There is also a related issue of how sign is determined for this metric. We don’t believe that the method for determining sign was explained in this paper, and we recommend making this adjustment. However, the signed l.e. LFC metric appears to be used in the figures. We assume that the sign is related to the sign of the next closest topic. But we are not sure how the definition occurs in the Bayesian setting of multiple states. In any case, it seems hard to determine a sign in cases such as the example above for topic B.

Our response. First, we would like to clarify any possible misunderstanding here: *the least extreme (l.e.) LFC is a signed metric.* To clarify, we review the definition from (6) and (7) in the paper. We start with the *pairwise LFC*, which is simply the LFC that compares the expression between topics *k* and *l*:

(1) LFC*_k,l_*(*j*) := log_2_*p_jk_*

*p_jl_.*

The l.e. LFC for topic *k*, denoted LFC^l^*^.^*^e^*^.^*

*_k_*^(^*^j^*^), is a pairwise LFC (1) in which the topic appear^ ing in the denominator, topic *l*, is chosen to be the topic that produces the *smallest change*: LFC^l^*^.^*^e^*^.^*

*_k_*(*j*) := LFC*_k,l_*(*j*) 

(2)

such that *l* = argmin*_l_^0^_6_*_=_*_k_|*LFC*_k,l_0* (*j*)*|,* 

Note that the absolute value is only inside the argmin, which determines *l*, whereas the l.e. LFC retains the sign. We have added some clarification to the text in the hopes that it will help with understanding this key point: “In words, the l.e. LFC for topic *k* is the LFC comparing topics *k* and *l*, in which *l* is chosen to be topic that results in the smallest change (hence ‘least extreme’).”

In the paper, we focus on identifying “distinctive genes”—a distinctive gene is one in which its expression is significantly different from its expression *in all other topics.* Typically, this will be a gene that is strongly overexpressed or strongly underexpressed in one topic. The reviewer’s example, with topics A, B and C, is an example in which this approach may not work so well, and one might prefer a different approach to measuring LFC. We have noted in the Methods (“Estimation of log-fold change”) that the GoM DE analysis framework is quite general, and in some settings one may prefer approaches other than the l.e. LFC. Two alternatives to the l.e. LFC are implemented in fastTopics R package.

Comment 3: Comparison to frequentist method. While we believe DE across topics to be a worthwhile endeavor, we believe that the paper would be strengthened by better moti vating the use of a Bayesian method for computing l.e. LFC. For example, one can consider simpler DE metrics computed directly based off the MLE or based off of MLE + fisher infor mation matrix. It would be great if the authors could provide additional analyses that clearly demonstrate the necessity of the Bayesian approach.

Our response. We believe the reviewer’s comment about the use of a “Bayesian method” is mainly about the choice of computational approach—in particular, the use of MCMC instead of a less computationally burdensome approach, for example, as the reviewer suggested, a method based on a Laplace (normal) approximation to the likelihood at the MLE. Indeed, the approach that the reviewer suggested would be the preferred approach, and it is in fact the first approach we implemented. This approach is also convenient as it is already implemented by the glm function in R with family = poisson(link = “identity”). However, two factors led us to consider a better approach.

The first factor was that confidence intervals for the model parameters *p_jk_* were sometimes severely underestimated, leading to false-positive DE genes. The reason for this was the inadequacy of the normal approximation in some cases, in particular for genes with very low expression. (The inadequacy of the normal approximation has been the topic of several recent papers on statistical methods for genome-wide association studies, e.g., Dey et al. 2017.) It is unclear to us why this issue hasn’t been mentioned or discussed in the differential expression analysis literature, at least as far as we have seen, but this appears to be an underappreciated or under-discussed issue.

The second factor was that our ultimate goal was to obtain measures of uncertainty for the main quantities of interest—the l.e. LFCs—and it was unclear how to translate the analytical computations of confidence intervals for the *p_jk_*’s to confidence intervals for the l.e. LFCs without making further approximations or simplifications. Consider that the l.e. LFCs do not have a simple linear relationship to the expression levels *p_jk_*, but instead involve an argmin operation, which makes analytical solutions particularly difficult to come by.

For these two reasons, we implemented an MCMC approach. We believe that more com putationally efficient (and possibly more accurate) solutions exist, but an advantage of the MCMC algorithm is that it was simple to implement, it is assumption-free, and it has the side benefit that it easily accommodates different choices of LFC (other options besides the l.e. LFC are also implemented in the R package). Please also see our response to Comment 2 which is related to this point.

In the revised manuscript,we have added some background at the beginning of “Quantify ing uncertainty and stabilizing LFC estimates” to better motivate the MCMC algorithm.

Comment 4: Length of paper / number of datasets. Since this is primarily a computational methods paper, and *Genome Biology* is a journal targeted at evaluating new methods, we wonder if the presentation of the biology of multiple datasets goes beyond what is necessary and could be reduced. Figures 6, 7, and 8 feel a little repetitive with some of the earlier figures, and we wonder if some can be moved to the supplement. In particular, the ATAC-seq data is probably the most interesting to focus on because it is application to a new type of data. We leave this as a suggestion for the discretion of the editor and the authors.

Our response. We compared the length of our paper to a small sampling of recent methods papers published in *Genome Biology.* Due to recent changes in formatting, we looked only at methods papers in the earlier two-column format (2019 and earlier). The lengths of these papers (not including Methods and text after that) ranged from 8 to 13 pages. By comparison, our paper is 12 pages (not including Methods and text after that). Following the the Editor’s advice, we decided not to move any more results to the supplement.

Minor comment 1. Appears to be no benchmark for runtime. For instance, how long did it take to obtain the results for Figure 1?

Our response. Thank you for pointing out this omission. We have added a new table (Ta ble 1) giving the GoM DE analysis running times for all the single-cell data sets.

Minor comment 2. Page 14 line 45, add definition of highest posterior density (HPD) inter val.

Our response. Formally defining an HPD interval is quite technical, and would involve in troducing other notation, which is why we referred to Chen and Shao (1999); Box and Tiao (1992) for the definition. To provide some intuition for the HPD interval, we have added the following detail to the revised manuscript: “To provide a reasonable substitute summarizing uncertainty in the estimates, we computed Monte Carlo estimates of highest posterior density (HPD) intervals. A (1 *− α*) HPD interval is the smallest interval that contains 100(1 *− α*)% of the probability mass (Chen and Shao, 1999; Box and Tiao, 1992).”

Minor comment 3. For the analysis on simulated data (page 4, line 38) the number of cells is only 200, which is significantly smaller than typical single-cell RNA-seq data.

Our response. We made a mistake in describing the simulations—for the simulations with 2 topics, we simulated 200 cells; for the simulations with 6 topics, we simulated 1,000 cells. We have corrected this mistake in the revised manuscript.

While the number of cells in these simulations is smaller than a typical current single cell data set, our intention was to test the methods in a more challenging scenario where differentially expressed genes would be more difficult to identify accurately due to the small sample size, and where it would be important to correctly quantify uncertainty to correctly control for false discovery rates.

Additionally, it is not unusual for a large single-cell data set to feature a rare cell type with a small number of cells, and therefore our simulations are reflective of this more challenging situation. For example, in the first set of simulations with 2 topics representing 2 discrete cell types, each cell type had 100 cells. Compare this to the scRNA-seq data set from Montoro et al. (2018) with 7,193 cells: two of the cell types (basal, club) were very abundant, with over 2,000 cells for each abundant cell type, whereas other cell types (e.g., goblet, ionocyte) were very rare, with fewer than 100 cells. (Note these numbers are estimates.)

In the text, we added a brief explanation for our simulation choices: “Note that although half the simulated genes had different expression levels in the two topics, most of these expression differences were small, and therefore we did not expect the methods to be able to identify most expression differences. This mimics the typical situation in gene expression studies whereby most expression differences are small.”

Minor comment 4. On Page 12, the section “Two group model” introduces new notation that could have been described using equation 9. Also, it appears that this model is never actually used in any other parts of the paper.

Our response. We agree that the two-group model is not essential to the paper, and might be confusing, and therefore we have removed it from the paper.

Minor comment 5. For Figure 2, should include an equation (in Methods or Results) for the posterior *z-*score.

Our response. The posterior *z*-score was defined in the Methods (“Quantifying uncertainty and stabilizing LFC estimates”) as the posterior mean divided by the posterior standard error. In the revised manuscript, we have included this definition for the Figure 2 caption

Minor comment 6. Is this paper looking for DE across topics, or looking for changes in topic-level expression across populations? Should make this distinction more clearly at the start. I believe that it only does DE across topics.

Our response. A key idea underlying this work is that the topics *are* the populations, or at least the topics should identify subpopulations of cells when they are clearly distinguishable from the data. This idea was first mentioned in the introduction (“Background”), where we wrote, “Parts-based representations share some of the features of both a clustering and a di mensionality reduction: on the one hand, they learn a lower dimensional representation of the cells; on the other hand, the individual dimensions (the “parts”) of the reduced representation can identify discrete clusters or discrete subpopulations.”

Minor comment 7. We recommend citing the paper “Cell type-specific inference of differ ential expression in spatial transcriptomics” (*Nature Methods*, 2022) as a similar model in a different context.

Our response. Thank you for pointing us to this paper. Indeed, although the aims of C-SIDE are different from ours, their model is closely related, and we have now included a citation to this paper.

Minor comment 8. Figure 1c: enrichment coefficients are less than 1. Are these in log space? If so, this could be more clearly clarified.

Our response. An explanation of the enrichment coefficients was provided in the Methods, but we agree that this explanation could have been made more accessible to the reader. We have now added an explanation to the caption for Figure 1: “An enrichment coefficient is an estimate of the expected increase in the LFC for genes that belong to the gene set relative to genes that do not belong to the gene set.” More detail about how these enrichment coefficients are obtained is provided in the Methods (“Gene set enrichment analysis”).

Minor comment 9. Typo: “genes upregulated in CD34+ in hematopoetic cells”. Our response. Thank you for pointing out this typo.

Minor comment 10. Figure 2: missing color scale?

Our response. The color scale is given in the top-right plot of Figure 2. It is the same for all plots in this figure.

Minor comment 11. Can the l.e. LFC metric also be applied to single-cell RNA-seq data? Has it been applied before? If so, how well does it work?

Our response. The l.e. LFC could potentially be used in other analyses of RNA-seq data, for example, in analyzing multi-condition single-cell RNA-seq data sets (Crowell et al., 2020; Liu et al., 2022). However, we are not aware of any studies where it has been used previously. One reason may be that it is more difficult to implement statistical inferences for the l.e. LFC (as we noted in our response to Comment 3 from Reviewer 1).

Minor comment 12. The notation in the two groups model in the methods section does not match the earlier notation. Authors should stick to the l (’ell’) variable or at least define the new variables in terms of the l variable. Also, the authors should state explicitly that they are considering discrete topics/non-admixed for the two groups model because we found this to be confusing.

Our response. This comment is related to an earlier comment (Minor comment 4). Given that the two-group model is not essential to the paper, and could potentially cause confusion, we have removed the two-group model from the paper.

Minor comment 13. “Maximum a posteriori estimation”: how are the gamma prior param eter values determined / motivated?

Our response. The gamma prior for *maximum a posteriori* (MAP) estimation was only in troduced to improve numerical stability of this estimation step, as we stated in the manuscript. The Gamma prior we used for all analyses in the paper was Gamma(*α, β*), with *α* = 1 + *ε*, *β* = 1, *ε* = 0*.*1. In practice, because this prior is “flat”, this will yield *maximum a posteriori* (MAP) estimates that are very close to maximum-likelihood estimates except in edge cases (e.g., for genes with very low expression).

Minor comment 14. Some of the estimated DE values are extremely large (13–15). It would be good to present some plots based on the raw data that can convince the reader that these are real effects present in the data, rather than an artifact of the topic learning procedure.

Our response. It is not unusual to obtain large LFC estimates; typically these are genes that uniquely or nearly uniquely expressed in the cell type. To take one recent example from the *Tabula Sapiens* paper (Jones et al., 2022), many LFC estimates for large intestine vs. small intestine (Figure 6E) were larger than 10. For example, the LFC estimate for gene *RPS19* was 19.16. Note the figure in that paper is misleading because the x-axis was truncated to [*−*5*,* 5]; the actual LFC estimates were given in Table S8.

REFERENCES

ARGELAGUET, R., VELTEN, B., ARNOL, D., DIETRICH, S., ZENZ, T., MARIONI, J. C., BUETTNER, F., HU BER, W. and STEGLE, O. (2018). Multi-omics factor analysis—a framework for unsupervised integration of multi-omics data sets. *Molecular Systems Biology* 14.

ARGELAGUET, R., ARNOL, D., BREDIKHIN, D., DELORO, Y., VELTEN, B., MARIONI, J. C. and STE GLE, O. (2020). MOFA+: a statistical framework for comprehensive integration of multi-modal single-cell data. *Genome Biology* 21 111.

ASHUACH, T., REIDENBACH, D. A., GAYOSO, A. and YOSEF, N. (2022). PeakVI: a deep generative model for single-cell chromatin accessibility analysis. 2 100182.

BOX, G. E. P. and TIAO, G. C. (1992). *Bayesian inference in statistical analysis*. Addison-Wesley, Reading, MA.

CAO, J., CUSANOVICH, D. A., RAMANI, V., AGHAMIRZAIE, D., PLINER, H. A., HILL, A. J., DAZA, R. M., MCFALINE-FIGUEROA, J. L., PACKER, J. S., CHRISTIANSEN, L., STEEMERS, F. J., ADEY, A. C., TRAP NELL, C. and SHENDURE, J. (2018). Joint profiling of chromatin accessibility and gene expression in thou sands of single cells. *Science* 361 1380–1385.

CHEN, M.-H. and SHAO, Q.-M. (1999). Monte Carlo estimation of Bayesian credible and HPD intervals. *Journal of Computational and Graphical Statistics* 8 69–92.

CROWELL, H. L., SONESON, C., GERMAIN, P.-L., CALINI, D., COLLIN, L., RAPOSO, C., MALHOTRA, D. and ROBINSON, M. D. (2020). muscat detects subpopulation-specific state transitions from multi-sample multi condition single-cell transcriptomics data. *Nature Communications* 11 6077.

DEY, K. K., HSIAO, C. J. and STEPHENS, M. (2017). Visualizing the structure of RNA-seq expression data using grade of membership models. *PLoS Genetics* 13 e1006599.

DEY, R., SCHMIDT, E. M., ABECASIS, G. R. and LEE, S. (2017). A fast and accurate algorithm to test for binary phenotypes and its application to PheWAS. *American Journal of Human Genetics* 101 37–49. GONZÁLEZ-BLAS, C., MINNOYE, L., PAPASOKRATI, D., AIBAR, S., HULSELMANS, G., CHRISTIAENS, V., DAVIE, K., WOUTERS, J. and AERTS, S. (2019). cisTopic: cis-regulatory topic modeling on single-cell ATAC seq data. *Nature Methods* 16 397–400.

HAO, Y., STUART, T., KOWALSKI, M. H., CHOUDHARY, S., HOFFMAN, P., HARTMAN, A., SRIVASTAVA, A., MOLLA, G., MADAD, S., FERNANDEZ-GRANDA, C. and SATIJA, R. (2023). Dictionary learning for inte grative, multimodal and scalable single-cell analysis. *Nature Biotechnology*. https://doi.org/10.1038/s41587- 023-01767-y

JIN, S., ZHANG, L. and NIE, Q. (2020). scAI: an unsupervised approach for the integrative analysis of parallel single-cell transcriptomic and epigenomic profiles. *Genome Biology* 21 25.

JONES, R. C., KARKANIAS, J., KRASNOW, M. A., PISCO, A. O., QUAKE, S. R., SALZMAN, J. et al. (2022). The Tabula Sapiens: a multiple-organ, single-cell transcriptomic atlas of humans. *Science* 376. LEVSKY, J. M. and SINGER, R. H. (2003). Gene expression and the myth of the average cell. *Trends in Cell Biology* 13 4–6.

LIU, Y., CARBONETTO, P., TAKAHAMA, M., GRUENBAUM, A., XIE, D., CHEVRIER, N. and STEPHENS, M. (2022). A flexible model for correlated count data, with application to analysis of gene expression differences in multi-condition experiments. *arXiv* 2210.00697.

MA, S., ZHANG, B., LAFAVE, L. M., EARL, A. S., CHIANG, Z., HU, Y., DING, J., BRACK, A., KARTHA, V. K., TAY, T., LAW, T., LAREAU, C., HSU, Y.-C., REGEV, A. and BUENROSTRO, J. D. (2020). Chromatin potential identified by shared single-cell profiling of RNA and chromatin. *Cell* 183 1103–1116.e20.

MONTORO, D. T., HABER, A. L., BITON, M., VINARSKY, V., LIN, B., BIRKET, S. E., YUAN, F., CHEN, S., LEUNG, H. M., VILLORIA, J., ROGEL, N., BURGIN, G., TSANKOV, A. M., WAGHRAY, A., SLYPER, M., WALDMAN, J., NGUYEN, L., DIONNE, D., ROZENBLATT-ROSEN, O., TATA, P. R., MOU, H., SHIV ARAJU, M., BIHLER, H., MENSE, M., TEARNEY, G. J., ROWE, S. M., ENGELHARDT, J. F., REGEV, A. and RAJAGOPAL, J. (2018). A revised airway epithelial hierarchy includes CFTR-expressing ionocytes. *Na ture* 560 319–324.

MORRIS, S. A. (2019). The evolving concept of cell identity in the single cell era. *Development* 146. SHIGA, M., SENO, S., ONIZUKA, M. and MATSUDA, H. (2021). SC-JNMF: single-cell clustering integrating multiple quantification methods based on joint non-negative matrix factorization. *PeerJ* 9 e12087. STUART, T. and SATIJA, R. (2019). Integrative single-cell analysis. *Nature Reviews Genetics* 20 257–272. YANG, Z. and MICHAILIDIS, G. (2015). A non-negative matrix factorization method for detecting modules in heterogeneous omics multi-modal data. *Bioinformatics* 32 1–8.

ZENG, Y., WEI, Z., ZHONG, F., PAN, Z., LU, Y. and YANG, Y. (2022). A parameter-free deep embedded clustering method for single-cell RNA-seq data. *Briefings in Bioinformatics* 23 bbac172. ZHANG, S., LIU, C.-C., LI, W., SHEN, H., LAIRD, P. W. and ZHOU, X. J. (2012). Discovery of multi dimensional modules by integrative analysis of cancer genomic data. *Nucleic Acids Research* 40 9379–9391.

**2^nd^ round**

**Reviewer 1**

I have no further questions

**Reviewer 2**

The authors have thoroughly addressed our comments and concerns. The authors have adequately addressed the most significant questions that we had about the manuscript. Below are some additional minore comments that will improve the manuscript further.

1. The definition of least extreme LFC should be motivated through a simple example, graphic, or table. I.e., a real (or hypothetical) gene with pairwise log fold changes and the corresponding least extreme LFC. This would benefit readers with less quantitative background to better understand the metric.

2. In pg.5 of the response, the authors agree that our hypothetical example with topics A,B, and C are an example where the proposed leLFC metric does not perform well. Although we agree that “distinctive genes” are of interest, how would the proposed approach be able to filter out a problematic gene like this one? Similarly, how would a user know if another metric (those provided by fastTopics) should be used instead of leLFC?

**Authors’ response**

Reviewer #2 requested (a) a simple example to illustrate the use of the "least extreme" LFC, and (b) a more detailed discussion on the use of the least extreme LFC. We have addressed both (a) and (b) in the revised manuscript.
